# Exploring the relationship between family support and eating behaviors among college students with subjective well-being as the central mediating variable

**DOI:** 10.3389/fpsyg.2026.1735152

**Published:** 2026-04-07

**Authors:** Cong Xue, Haiou Peng, Geng Li, Juyan Fang, Xingrui Wang, Binbin Zhao, Pingfan Liu, Zhiru Liang, Wenxi Tang

**Affiliations:** 1 Jishou University, Jishou, China; 2 Hunan University of Medicine, Huaihua, China; 3 School of Psychology, Research Center for Exercise and Brain Science, Shanghai University of Sport, Shanghai, China; 4 School of Physical Education, Xichang University, Xichang, China; 5 College of Sports Science, Shenyang Normal University, Shenyang, China; 6 Department of Hepatobiliary Surgery, Xilingol League Central Hospital, Xilinhot, Inner Mongolia, China

**Keywords:** anxiety, depression, eating behaviors, family support, subjective wellbeing, university students

## Abstract

**Background and objective:**

Family support is widely recognized as a critical social resource that significantly shapes individuals’ mental health and lifestyle choices. However, the extent to which it indirectly influences eating behaviors through psychological mechanisms remains insufficiently understood. Although existing literature has highlighted the pivotal role of familial support in the formation of health-related behaviors, the underlying pathways through which this influence is exerted have not been systematically elucidated. The present study seeks to clarify the chain-mediating mechanism by which familial support affects university students’ dietary habits via negative emotional states and subjective wellbeing. By doing so, this research aims to provide both theoretical insights and practical guidance for the development of psychological adjustment strategies and behavioral interventions.

**Methods:**

A convenience sampling strategy was adopted to recruit 3,007 undergraduates in 2024 for a self-report survey, including 1,267 males and 1,740 females, with an average age of 19.03 years (SD = 1.176). The study applied a cross-sectional design and employed validated measurement tools, such as the Family support Scale, Anxiety Scale, Depression Scale, Subjective wellbeing Scale, and Healthy Eating Behaviors Scale. Correlational analyses were performed to assess the relationships among the core variables. Mediation effects were further examined using the PROCESS macro in SPSS to test the proposed pathways of the model.

**Results:**

Family support demonstrated a negative correlation with anxiety (*r* = −0.210) and depression (*r* = −0.211), while showing positive associations with subjective wellbeing (*r* = 0.250) and healthy eating behaviors (*r* = 0.168). Anxiety was inversely related to both subjective wellbeing (*r* = −0.378) and healthy eating (*r* = −0.206), whereas depression was also negatively linked to subjective wellbeing (*r* = −0.386) and healthy eating (*r* = −0.210). Additionally, subjective wellbeing exhibited a positive correlation with healthy eating (*r* = 0.185). Further analysis revealed a significant chain mediation effect of negative emotional states and subjective wellbeing in explaining the relationship between family support and healthy eating among university students. Furthermore, family support was significantly positively associated with eating behaviors (*β* = 0.610, *p* < 0.001), whereas anxiety (*β* = −0.344, *p* < 0.001), depression (*β* = −0.050, *p* < 0.001), and subjective wellbeing (*β* = 0.327, *p* < 0.001) were significantly negatively associated with eating behaviors. After the inclusion of mediating variables, this effect remained significant (*β* = 0.401, *p* < 0.001). Meanwhile, subjective wellbeing was significantly positively associated with eating behaviors (*β* = 0.185, *p* < 0.001). Finally, anxiety (*β* = −0.387, *p* < 0.001) and depression (*β* = −0.411, *p* < 0.001) were significantly negatively associated with subjective wellbeing, and both anxiety (*β* = −0.177, *p* < 0.001) and depression (*β* = −0.217, *p* < 0.001) showed negative associations with eating behaviors.

**Conclusion:**

This study further elucidates the underlying mechanisms by which family support contributes to the enhancement of healthy eating behaviors among university students, specifically by identifying the indirect pathways mediated by negative emotional states. The findings demonstrate that anxiety and depression serve as significant mediators in the association between family support and eating behaviors. These results not only enrich the theoretical understanding of how social support influences health-related behaviors but also offer robust empirical evidence and strategic implications for universities aiming to integrate psychological wellbeing promotion with lifestyle interventions.

## 1 Background

Family support (FS) received during university years exerts a profound influence on students’ eating behaviors, while irregular eating patterns or unhealthy food choices may, in turn, heighten the risk of developing depressive and anxious symptoms. Eating behaviors among university students refers to their consistent patterns of food selection, portion control, and meal timing in daily life. These behaviors not only reflect nutritional intake but also serve as indicators of students’ lifestyle regularity and health consciousness (Sogari et al., 2018). Disordered eating patterns and binge eating are particularly prevalent among young people, especially adolescents and university students (Vila-Martí et al., 2021). A cluster analysis conducted in 2015 involving 345 undergraduates utilized a self-reported “Student Eating Behaviors” questionnaire to examine the consumption prevalence of snacks, convenience store foods, and fast food (excluding fruits and vegetables). The findings indicated that only 19% of participants were classified as engaging in “healthy eating behaviors” (Tanton et al., 2015). A cross-sectional study further revealed that approximately 28.2% of university students exhibited varying degrees of disordered eating behaviors, with 40.2% reporting episodes of binge eating (Ganson et al., 2022). University years are widely recognized as a critical period for the establishment of long-term healthy dietary patterns. However, in reality, due to the combined effects of financial constraints, academic stress, poor time management, and insufficient nutritional knowledge, university students are particularly vulnerable to developing poor eating habits (Almoraie et al., 2024). A 2015 study targeting 244 female undergraduates found that 48.8% reported symptoms indicative of disordered eating (Ko et al., 2015). Among this population, poor eating behaviors and body image dissatisfaction are increasingly prevalent and are closely associated with heightened risks of chronic illnesses such as cardiovascular disease and diabetes. Moreover, these behaviors pose substantial threats to mental health, often manifesting as social anxiety and depressive symptoms (Almoraie et al., 2024). In summary, systematically identifying and exploring the underlying determinants of university students’ eating behaviors holds significant practical value. Such research not only contributes to the identification of critical risk factors linked to abnormal eating but also provides a robust theoretical foundation and strategic direction for the development of evidence-based health promotion interventions in university settings.

University students, situated at a critical juncture of psychological and behavioral development, exhibit heightened sensitivity and vulnerability. Upon entering university, they are confronted with a series of challenges brought on by unfamiliar environments and shifting social roles—challenges that are especially pronounced among those who live independently and away from their families, where the absence of daily parental supervision and emotional support can increase the likelihood of adopting maladaptive eating behaviors (Bárbara and Ferreira-Pêgo, 2020). In this context, FS emerges as a multidimensional construct encompassing emotional care, instrumental assistance, and informational guidance, with its core rooted in reciprocal interactions and supportive dynamics among family members. Such mechanisms serve a salient regulatory function, exerting a positive influence on individuals’ psychological states and behavioral choices when confronted with external stressors (Kamaryati and Malathum, 2020). Grounded in social cognitive theory, human behavior is posited to be shaped by the reciprocal interplay between internal psychological mechanisms—such as self-efficacy and goal orientation—and external environmental influences, including social support and situational factors, both of which play integral roles in the initiation and maintenance of eating behaviors (Doerksen and McAuley, 2014). Empirical evidence has substantiated that both internal motivations and external conditions significantly inform behavioral decision-making in the realm of dietary practices. Within familial contexts, university students frequently internalize dietary cognitions by observing the eating patterns, attitudes, and lifestyle habits of their parents or other household members, gradually assimilating these elements through routine interaction, observational learning, and behavioral reinforcement. A cross-sectional study conducted in 2023, encompassing 702 university students across 29 provinces in China, further illuminated the pivotal role of family cohesion in the development of eating behaviors among this demographic. The findings revealed a statistically significant positive correlation between family cohesion and healthy dietary practices, thereby underscoring the direct impact of familial dynamics on behavior formation (Yang et al., 2023). The ecological model, adopting a multilayered analytical perspective, links individual behavior to the broader social environment and structural determinants, positing that behavioral outcomes are not solely a reflection of intrinsic attributes but are also shaped by interpersonal relationships and contextual surroundings. Within this framework, both supportive behaviors from family members and social pressures from peer groups are regarded as critical external determinants of dietary decision-making. While FS typically fosters the maintenance of health-promoting behaviors, negative peer influence may precipitate unhealthy eating habits (Sallis and Owen, 2015). An empirical investigation conducted in 2013 involving 746 American university students corroborated the promotive function of familial support, indicating that higher perceived FS was associated with a 14% increase in the likelihood of daily fruit and vegetable consumption, alongside a 50% rise in the proportion of individuals engaging in at least 30 min of moderate-to-vigorous physical activity per day (Students’ Diet and Physical Activity Improve with Parent Communications, n.d.). Taken together, these findings provide a robust foundation for the present study’s hypothesis that FS is positively correlated with healthy eating behaviors.

University students, situated at a pivotal stage of psychological maturation and social adaptation, are particularly susceptible to the detrimental effects of prolonged negative emotional states, which may trigger a cascade of adverse outcomes such as diminished academic performance, strained interpersonal relationships, and, in more severe instances, the onset of psychological disorders including anxiety and depression—thereby posing substantial threats to both their mental wellbeing and educational trajectory (Mu et al., 2024). Negative emotions are typically characterized by an individual’s adverse affective responses and corresponding behavioral manifestations in reaction to external stimuli, encompassing a spectrum of distressing psychological states such as tension, sadness, fear, guilt, anger, contempt, and disgust. A meta-analysis conducted in 2018, which synthesized data from 1,841 university students across Qatar and Lebanon, reported prevalence rates of depression (PHQ-9 ≥ 12) and generalized anxiety disorder (GAD-7 ≥ 10) at 34.6 and 36.1%, respectively—figures notably higher than the corresponding rates observed in U.S. college populations, which stood at 12.8 and 15.9% (Kronfol et al., 2018). Additionally, a cross-sectional study involving 1,899 students from France (*n* = 1,094), Moldova (*n* = 268), and Romania (*n* = 537) revealed depression and anxiety prevalence rates of 39.0 and 47.0%, respectively (Habihirwe et al., 2018). Both attachment theory (Scharfe, 2021) and emotion regulation theory (McRae and Gross, 2020) underscore the critical role of parental attachment in shaping an individual’s psychological development, daily functioning, and emotional expression. Secure and supportive parent–child attachment relationships foster heightened emotional security and coping capacity, thereby enhancing resilience to stress and the ability to regulate negative emotions. Moreover, individual attachment styles have been shown to significantly influence emotional states and subsequent behavioral decisions, including those related to dietary choices. Within this framework, FS represents a vital external resource for emotional regulation among university students, particularly in contexts of social withdrawal or perceived isolation; students who perceive higher levels of familial support are generally better equipped to maintain mental stability (Yang et al., 2022). However, when individuals fail to alleviate anxiety through certain coping behaviors, they may descend into deeper emotional distress. From a cognitive processing perspective, anxious university students often exhibit notable cognitive biases, such as an excessive focus on internal emotional experiences and a pronounced tendency to seek temporary emotional relief through the consumption of high-calorie, sugar-rich foods (Rosenbaum and White, 2013). According to the cognitive appraisal theory of stress, the availability and perceived adequacy of FS—as a critical external resource—exerts a significant influence on students’ psychological states, suggesting a moderate inverse relationship between FS and anxiety levels (Liu et al., 2024a; Liu et al., 2025a; Liu et al., 2025b). As a pervasive psychological condition, anxiety not only compromises individual mental health directly but also affects behavioral patterns through various psychological mechanisms (Liu et al., 2024b; Wang et al., 2025a; Wang et al., 2025b; Wang et al., 2025c), particularly in the domain of eating behaviors (Hussenoeder et al., 2021). A meta-analysis conducted in 2019 involving a sample of 1,589 university students explored the predictive role of anxiety sensitivity in dietary expectations and revealed that students with elevated anxiety sensitivity were more likely to develop maladaptive expectations regarding food intake, thereby heightening their susceptibility to unhealthy eating behaviors (Kauffman et al., 2021). Similarly, a cross-sectional study investigating the relationship between emotional states and eating behaviors among university students found that individuals with higher levels of depression reported significantly greater saturated fat intake and lower consumption of fruits and vegetables, reinforcing the proposition that emotional disorders may exacerbate the formation of detrimental dietary patterns (Keck et al., 2020). Drawing upon the aforementioned empirical findings, the present study posits that negative emotions mediate the relationship between FS and healthy eating behaviors.

The eating behaviors of university students are not only influenced by external environmental factors such as familial support but are also profoundly shaped by the internal psychological regulatory mechanism of subjective wellbeing (SWB). SWB refers to an individual’s overall cognitive evaluation of their life quality, encompassing both affective experiences and cognitive assessments of life satisfaction (Proctor, 2014). Within the theoretical framework of the buffering hypothesis, social support and other psychological resources serve as protective factors that can mitigate or even counteract the detrimental impact of stress-inducing events (Jensen-Fielding, 2019). Specifically, FS, as a critical form of social support, exerts a pronounced buffering effect on the psychological wellbeing of university students when they are confronted with academic, social, or personal stressors. Empirical findings have demonstrated that academic stress is directly associated with depressive symptoms and may further compromise mental health by impairing emotional regulation and sleep quality. Importantly, SWB has been shown to moderate these associations, particularly by attenuating the mediating effect of negative emotions in the pathway from negative emotions to sleep disturbances (Peng et al., 2025; Liu et al., 2023; Yin et al., 2025). A study conducted in 2022 involving 679 students from Hashim University employed cluster analysis and revealed a significant positive correlation between tangible support provided by family and students’ levels of SWB. Notably, 20% of the sample reported low perceived familial support, underscoring the pivotal role of familial resources in enhancing both wellbeing and social adaptability (Mahasneh, 2022). According to the theory of planned behavior, the formation of future behavioral patterns is largely determined by behavioral intentions, which are influenced by several psychological constructs including attitudes toward the behavior, subjective norms, and perceived behavioral control (Kan and Fabrigar, 2017). In the context of eating behaviors among university students, if individuals maintain a favorable attitude toward healthy eating (e.g., acknowledging the benefits of whole grains), perceive positive dietary expectations from family or peers (i.e., subjective norms), and believe in their capacity to access nutritious food and implement appropriate dietary routines (i.e., perceived behavioral control), the likelihood of forming strong intentions and translating them into actual healthy eating behaviors is substantially enhanced (Wang, 2018). Therefore, SWB, influenced by both the degree of perceived FS and individual emotional regulation capacity, may exert an indirect effect on students’ dietary habits by modulating their behavioral intentions. Supporting this proposition, a 2015 study conducted across five public universities in Chile examined 369 university students using cluster analysis and found that those reporting higher levels of perceived familial support experienced fewer health issues, adhered to healthier dietary patterns, and expressed a greater appreciation for the role of nutrition in maintaining overall health (Schnettler et al., 2015). Drawing from social cognitive theory (Decan and Kan, 2024), university students often construct their eating behaviors patterns through observational learning, whereby they adjust their choices based on behaviors exhibited by others. These observational targets may include not only peers in their immediate social environment but also individuals in virtual contexts such as social media platforms. Consequently, such exposure may encourage increased consumption of fruits, vegetables, whole grains, high-quality proteins, and beneficial fats, while simultaneously reducing the intake of sugars, saturated fats, processed foods, and refined carbohydrates—thereby contributing to both physical health and psychological wellbeing (An et al., 2024). In summary, it is hypothesized that SWB mediates the relationship between FS and eating behaviors among university students, offering a novel theoretical and empirical lens for elucidating the mechanisms underlying the formation of health-promoting behaviors.

Among university students, the formation and regulation of eating behaviors are not merely driven by direct familial support but are instead mediated through a series of psychological mechanisms, in which negative emotional states—such as anxiety and depression—and SWB play pivotal intermediary and bridging roles. The university stage marks a critical developmental transition from dependency to autonomy, during which students are commonly confronted with academic demands, interpersonal challenges, and the need for independent living, all of which render them particularly susceptible to emotional fluctuations. Depression, as a prevalent form of negative affect, not only compromises academic focus and task completion but may also exert enduring detrimental effects on both psychological and physiological wellbeing (Volkman et al., 2024). According to self-regulation theory (Begum, 2025), an individual’s ability to recognize and manage emotions constitutes a fundamental aspect of mental health. When students fail to effectively cope with anxiety or similar adverse experiences, they may exhibit maladaptive behaviors such as social withdrawal and impaired daily functioning. Conversely, the adoption of adaptive coping strategies and behavioral adjustments has been shown to alleviate emotional distress and significantly enhance SWB (Fu et al., 2024), thereby indicating a negative association between anxiety and SWB among university students. In alignment with social support theory (Leahy-Warren, 2014), early social relationships—particularly those involving family and peers—form the bedrock of an individual’s support network and exert a foundational influence on both mental health and behavioral development. When university students experience heightened anxiety, the presence of sufficient social support often facilitates the development of constructive psychological resources, thereby enhancing their coping capacity and behavioral self-regulation. This, in turn, promotes healthier dietary choices (Malone and Wachholtz, 2018). Moreover, as a core indicator of emotional regulatory capacity, higher levels of SWB further aid in modulating the disruptive influence of negative emotions on behavioral decisions, thereby contributing to the maintenance of a positive and health-oriented lifestyle (Disabato et al., 2021). On the basis of the aforementioned theoretical and empirical foundations, the present study posits the hypothesis that negative affect and SWB function as serial mediators in the relationship between FS and health-promoting eating behaviors among university students.

**Figure 1:**
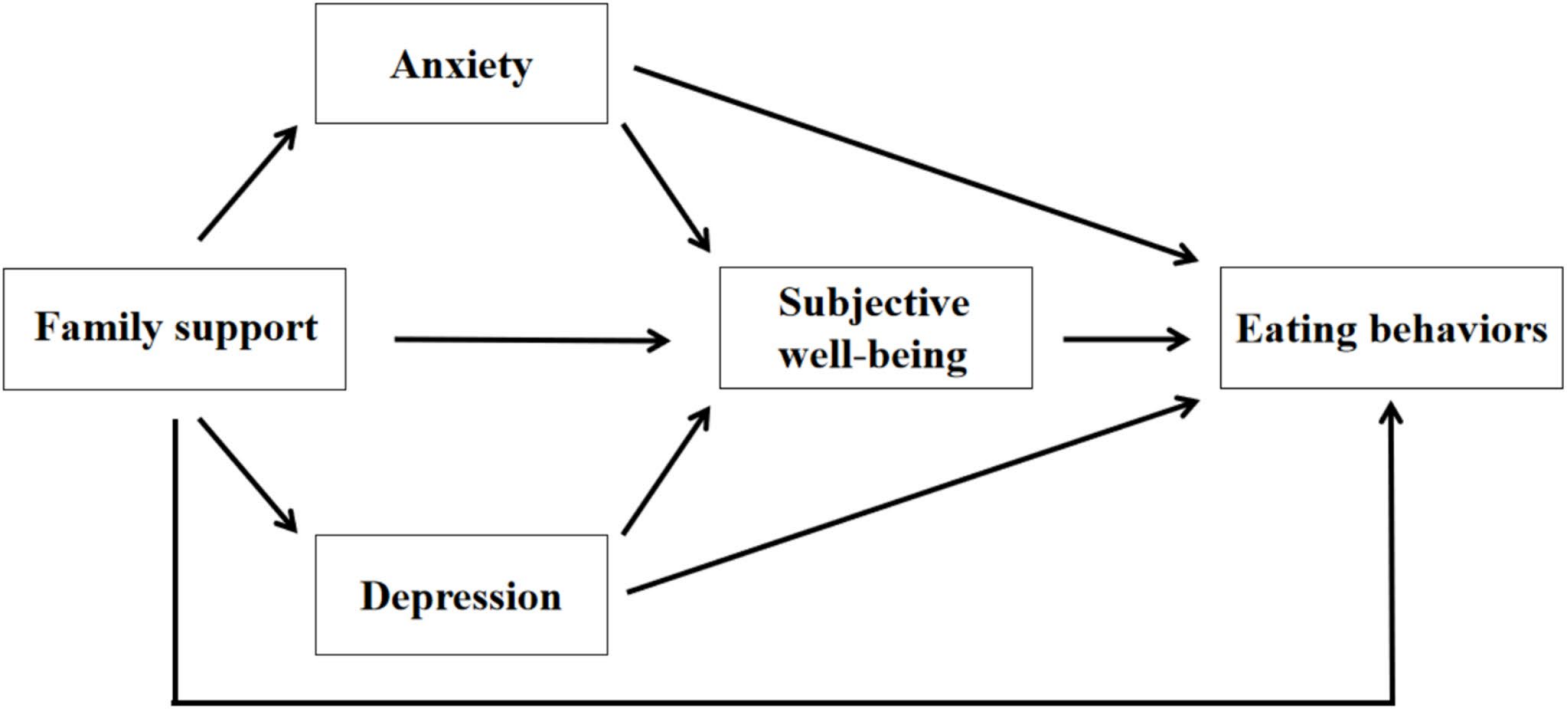
Chain hypothesized a mediation model.

Synthesizing prior research, it can be inferred that the level of perceived FS among university students may indirectly shape their engagement in healthy eating, with this process likely mediated by psychological and emotional variables. The present study seeks to explore the underlying relationship between FS and healthy eating within university populations, with specific attention to the mediating roles of negative emotions—namely anxiety and depression—and SWB. Existing literature consistently highlights the essential role of familial support in fostering students’ psychological adjustment and behavioral development, serving as a reliable source of emotional stability and security. Such support is crucial in reducing adverse emotional responses associated with academic, interpersonal, and life-related pressures. Conversely, insufficient FS is often linked to heightened anxiety and depressive symptoms, which may, in turn, diminish students’ SWB. A reduction in SWB can negatively impact both motivation and capacity to maintain health-conscious dietary practices. These findings suggest that FS influences healthy eating primarily through indirect pathways by shaping students’ emotional conditions and overall wellbeing. Within this framework, the current study introduces a chain mediation model to systematically test the mechanisms by which negative affect and SWB mediate the connection between FS and healthy eating. By empirically validating this model, the research aims to advance understanding of the dynamic interplay among psychological wellbeing, FS, and health-related behaviors in university students, while also providing theoretical insights and practical guidance for developing evidence-based mental health interventions and behavior promotion strategies in higher education settings (see Figure 1).

## 2 Methods

### 2.1 Participants

This study adopted a cross-sectional design. Based on the sample size calculation formula n = μa^2^ × [*π* × (1 − π)]/δ^2^, the minimum required sample size was estimated to be 385. Considering that cluster sampling was employed in this study, the required sample size was doubled to 770. In November 2024, a combination of cluster sampling and convenience sampling was used. Prior to the formal survey implementation, the research team first obtained informed consent from course instructors and then contacted teachers who were available to assist with questionnaire distribution (convenience sampling). Subsequently, the questionnaires were distributed by these teachers to all students in their respective classes (cluster sampling). To ensure regional representativeness and sample diversity, participants were recruited from universities located in four regions of China: North China, Central China, Southwest China, and South China. Prior to data collection, the study protocol was reviewed and approved by the Medical Ethics Committee of the authors’ affiliated institution (Approval No.: [JSDX-2025-0104]), ensuring compliance with all ethical and legal standards. All research procedures adhered to the ethical principles established by the institutional review board, thereby enhancing the reliability of the findings and fostering participant trust. The first page of the online questionnaire contained the informed consent statement and a brief description of the study, including information on data usage, complete confidentiality, and anonymization. Participants were allowed to proceed to the subsequent sections of the questionnaire only after reading the information and clicking “Agree.” If participants chose not to click “Agree” or withdrew from the survey at any point, their decision was fully respected, and no negative consequences were imposed, in accordance with participants’ rights and ethical principles. Completion time was approximately 5 min. Data screening excluded responses with unusually short completion times, repetitive patterns, or signs of inattentiveness. Prior to statistical analysis, the raw data were screened to ensure data quality. The inclusion criteria were as follows: (1) informed consent was obtained from the student prior to participation; (2) participants were enrolled undergraduate students; (3) participants were able to understand and independently complete the questionnaire; and (4) all items were completed with logically consistent responses. The exclusion criteria included questionnaires with overly stereotyped response patterns or abnormally short or long completion times. Specifically, responses were excluded if participants selected the same option consecutively for most items or exhibited repetitive, wave-like response patterns, as well as if the completion time fell beyond ±3 standard deviations from the mean response time. A total of 3,200 questionnaires were distributed and returned in this study. After cleaning, 3,007 valid responses remained, representing a 93.97% response rate. The sample consisted of 1,267 males and 1,740 females, with a mean age of 19.03 years (*M* = 19.03, SD = 1.176). Academic year distribution included 1,371 first-year, 1,431 s-year, 194 third-year, and 11 fourth-year students (*M* = 1.62, SD = 0.626).

### 2.2 Measurement tools

#### 2.2.1 Family support

The present study employed the FS Scale developed by Ng et al. (2010) and Ramaswamy et al. (2009). Participants responded to the statement: “My family listens to me when I speak.” Answers were recorded on a 5-point Likert scale from 1 (strongly disagree) to 5 (strongly agree). Higher scores represent stronger perceived FS, while lower scores indicate weaker support

#### 2.2.2 Negative emotions

This study used the Generalized Anxiety Disorder Scale (GAD-2) (Byrd-Bredbenner et al., 2021) and the Depression Questionnaire (PHQ-2) compiled by Levis et al. (2020) to assess the depressive symptoms of college students. The scale consists of two items, and the total score is scored using a 4-point Likert scale (1 = not at all, 4 = almost every day), with a total score of 2–8 points. The higher the score, the more severe the depressive and anxiety symptoms. In this study, the Cronbach’s *α* of the scales were 0.852 and 0.783, respectively. These measurement tool has been widely used in other studies (Yan et al., 2026; Yang et al., 2025).

#### 2.2.3 Subjective wellbeing

This study utilized a single-item measure (Huppert et al., 2009), to assess university students’ subjective wellbeing. The assessment was based on the following prompt: “If you were to comprehensively evaluate your current life, to what extent would you rate your overall happiness or unhappiness?” Responses were recorded on a 7-point Likert scale (1 = Completely unhappy; 7 = Completely happy), with higher scores indicating greater subjective wellbeing. These measure has been employed in previous research (Raudenská, 2023; Peng et al., 2025; Yin et al., 2025).

#### 2.2.4 Eating behaviors

This study employed three items to assess college students’ healthy eating habits. The assumptions are as follows: “Frequency of drinking soda, eating fruits (excluding juice), and vegetables (including salads and non-fried potatoes) over the past week.” Responses range from 0 (days) to 5 (5 days or more). The total score is obtained by adding the consumption of fruits and vegetables and subtracting the consumption of soda, with scores ranging from −5 to 10, where higher scores indicate healthier dietary habits (California Healthy Kids Survey (CHKS)|EdInstruments, n.d.). These measurement tool has been widely used in other studies (Yang et al., 2025).

### 2.3 Covariates

The analytical framework incorporated gender, age, academic year, only-child status, and parental education level as covariates, in order to account for potential confounding effects of demographic factors on the associations among core study variables, thereby strengthening the internal validity and explanatory power of the findings.

### 2.4 Statistical analysis

Data analysis began with calculating descriptive statistics and Pearson correlation coefficients for all key variables using SPSS 29.0 (Podsakoff et al., 2003). Before analyzing the main variables, the normality of the data was assessed using the Shapiro–Wilk test. Data were considered to approximate a normal distribution when the absolute values of skewness were less than 2 and the absolute values of kurtosis were less than 7 (Kim, 2013). The results indicated that the primary variables met the assumptions of normality, allowing the use of parametric statistical tests in subsequent analyses. To evaluate potential multicollinearity among variables, variance inflation factors (VIFs) were calculated. VIF values below 5 indicated that multicollinearity was not a concern in the present study (James et al., 2021). The hypothesized multiple mediation pathways were then tested using Model 80 in the PROCESS macro. Within the proposed analytical model, perceived FS was designated as the independent variable, eating behaviors was specified as the dependent variable, and both negative affect and SWB were incorporated as mediators. To enhance the robustness and precision of parameter estimation, a bias-corrected bootstrap method based on 5,000 resamples was employed to derive 95% confidence intervals for the indirect effects. This approach enabled an assessment of the mediating influence of negative emotional states on dietary outcomes across varying levels of SWB. All statistical inferences were conducted using two-tailed tests, with a significance threshold set at *p* < 0.05 (Berkovits et al., 2000).

## 3 Results

### 3.1 Common method bias test

Harman’s single-factor test was used to evaluate potential common method bias. An unrotated exploratory principal component analysis extracted four factors with eigenvalues greater than 1. The first factor explained 19.93% of the total variance, which is below the recommended threshold of 40%. These results indicate that common method bias did not significantly affect the data, supporting the internal validity of the measurements.

### 3.2 Descriptive analysis

As shown in Table 1, significant gender differences were observed in FS (*t* = −2.05, *p* < 0.05) and eating behaviors (*t* = −4.31, *p* < 0.001). Additionally, significant grade-level differences emerged for perceived FS (*F* = 3.67, *p* < 0.01), anxiety (*F* = 4.48, *p* < 0.001), depression (*F* = 5.15, *p* < 0.01), and SWB (*F* = 5.42, *p* < 0.001).

**Table 1:** Describes the analysis.

Variables		Family support		Anxiety		Depression		Subjective wellbeing		Eating behaviors	
		Mean	Sd	Mean	Sd	Mean	Sd	Mean	Sd	Mean	Sd
Gender	Boys	4.13	0.75	3.32	1.29	3.48	1.25	5.02	1.44	4.86	2.73
	Girls	4.19	0.74	3.39	1.16	3.54	1.12	5.05	1.34	5.29	2.78
	*t*	−2.05*		1.68		−1.46		−0.56		−4.31***	
Only child status	Only child	4.19	0.79	3.34	1.23	3.52	1.20	5.08	1.42	5.01	2.79
	Non-only child	4.16	0.73	3.36	1.22	3.52	1.17	5.03	1.37	5.13	2.76
	*t*	0.98		−0.40		0.46		−0.74		−0.97	
Grade	Freshman	4.19	0.73	3.29	1.14	3.45	1.09	5.10	1.32	5.25	2.80
	Sophomore year	4.13	0.75	3.45	1.28	3.60	1.24	4.96	1.44	4.95	2.76
	Junior	4.22	0.68	3.18	1.32	3.33	1.26	5.21	1.40	5.28	2.56
	Senior	3.67	1.66	3.00	0.87	3.44	1.33	5.44	0.88	5.78	2.54
	*F*	3.67**		4.48***		5.15***		5.42***		2.62	

*p < 0.05; **p < 0.01; ***p < 0.001.

### 3.3 Correlation analysis

As shown in Table 2, perceived FS was significantly negatively correlated with anxiety (*r* = −0.210, *p* < 0.001) and depression (*r* = −0.211, *p* < 0.001). In contrast, FS showed significant positive correlations with SWB (*r* = 0.250, *p* < 0.001), and eating behaviors (*r* = 0.168, *p* < 0.001). Anxiety was significantly negatively associated with SWB (*r* = −0.378, *p* < 0.001) and eating behaviors (*r* = −0.206, *p* < 0.001). Similarly, depression was negatively correlated with SWB (*r* = −0.386, *p* < 0.001) and eating behaviors (*r* = −0.210, *p* < 0.001). Additionally, a significant positive relationship was found between SWB and eating behaviors (*r* = 0.185, *p* < 0.001), indicating that individuals with higher SWB tend to adopt healthier eating patterns.

**Table 2:** Correlation analysis.

Variables	1	2	3	4	5
1. Family support	–				
2. Anxiety	−0.210***	–			
3. Depression	−0.211***	0.785***	–		
4. Subjective wellbeing	0.250***	−0.378***	−0.386***	–	
5. Eating behaviors	0.168***	−0.206***	−0.210***	0.185***	–

***p < 0.001.

### 3.4 Mediation model testing

**Figure 2:**
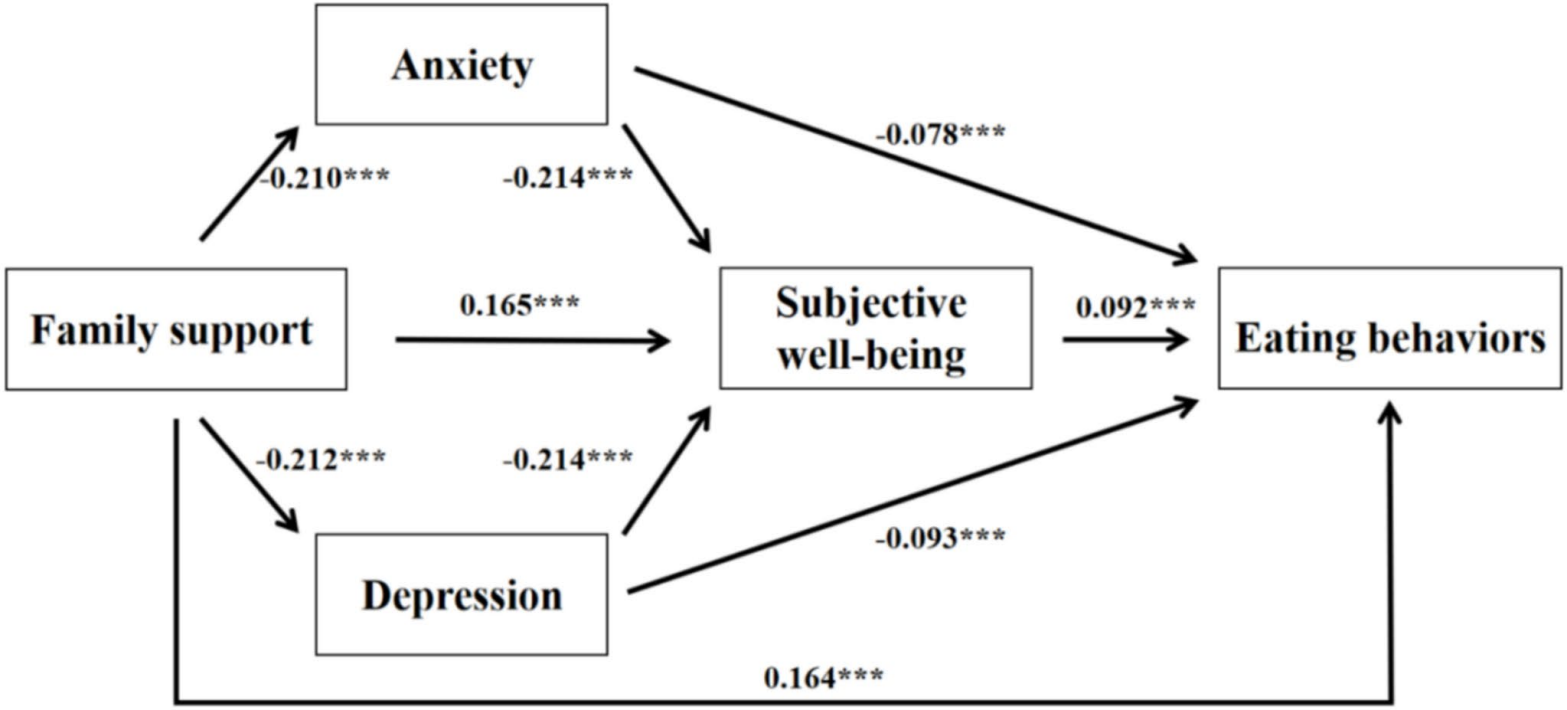
Chain mediation model.

After controlling for demographic variables, the results presented in Table 3 and Figure 2 indicate that family support was significantly and positively associated with eating behaviors (*β* = 0.164, *p* < 0.001). This association remained significant even after the inclusion of the mediating variables (*β* = 0.106, *p* < 0.001). In addition, family support was negatively associated with anxiety (*β* = −0.210, *p* < 0.001) and depression (*β* = −0.212, *p* < 0.001). Meanwhile, both anxiety and depression were significantly negatively associated with eating behaviors (anxiety: *β* = −0.078, *p* < 0.001; depression: *β* = −0.093, *p* < 0.001). Furthermore, family support showed a significant positive association with subjective wellbeing (*β* = 0.165, *p* < 0.001), and subjective wellbeing was positively related to eating behaviors (*β* = 0.092, *p* < 0.001). Finally, anxiety, depression, and subjective wellbeing were found to mediate the relationship between family support and eating behaviors among university students (anxiety: *β* = −0.175, *p* < 0.001; depression: *β* = −0.214, *p* < 0.001). The proportions of the mediating pathways are reported in detail in Table 4.

**Table 3:** Mediation model test.

Outcome variables	Predictor variables	*β*	SE	*t*	*R* ^2^	*F*
Eating behaviors	Family support	0.164	0.018	9.079***	0.034	17.833***
Anxiety	Family support	−0.210	0.018	−11.660***	0.046	24.230***
Depression	Family support	−0.212	0.019	−11.771***	0.047	24.425***
Subjective wellbeing	Family support	0.165	0.017	9.707***	0.191	88.664***
	Anxiety	−0.175	0.027	−6.561***		
	Depression	−0.214	0.029	−8.040***		
Eating behaviors	Family support	0.106	0.018	5.725***	0.078	27.976***
	Anxiety	−0.078	0.029	−2.722**		
	Depression	−0.093	0.028	−3.229***		
	Subjective wellbeing	0.092	0.020	4.746***		

**p < 0.01, ***p < 0.001.

**Table 4:** Mediation model path analysis.

Path	Effect size	SE	Bootstrap 95% CI	Proportion of mediating effect
Total effect	0.164	0.018	0.129, 0.200	
Direct effect	0.106	0.018	0.069, 0.142	
Total indirect effect	0.059	0.007	0.046, 0.073	35.97%
Family support → anxiety → eating behaviors	0.016	0.006	0.004, 0.029	9.76%
Family support → depression → eating behaviors	0.020	0.007	0.007, 0.033	12.19%
Family support → subjective wellbeing → eating behaviors	0.015	0.004	0.008, 0.023	9.15%
Family support → anxiety → subjective wellbeing → eating behaviors	0.003	0.001	0.002, 0.005	1.83%
Family support → depression → subjective wellbeing → eating behaviors	0.004	0.001	0.002, 0.007	2.44%

## 4 Discussion

The present study examined the relationship between perceived FS and eating behaviors among university students, with a specific focus on the mediating effects of negative emotions and SWB. Mediation analyses demonstrated that higher levels of perceived familial support were associated with more health-promoting dietary practices, both through direct pathways and indirectly via reductions in anxiety and depression as well as enhancements in SWB. Specifically, elevated FS was linked to diminished negative affect, which in turn contributed to improved levels of SWB, ultimately facilitating healthier dietary patterns. Negative emotions and SWB together served as partial mediators in the FS–eating behavior relationship, underscoring the important regulatory function of psychological states in shaping health-related lifestyle choices. These findings provide a more detailed understanding of the connections between psychological resources, emotional regulation, and health behavior in university students. They also offer theoretical and practical insights for interventions designed to improve both physical and psychological wellbeing in higher education contexts.

The results of this study demonstrate a significant positive correlation between perceived FS and healthy eating behaviors among university students, which is consistent with previous empirical findings and supports Hypothesis H1 (Alshehri et al., 2023). Under the favorable influence of familial support, students’ eating habits are reinforced both emotionally and behaviorally, while simultaneously being shaped by the deeper imprint of cultural capital embedded within the family context. Drawing upon Cultural Capital Theory (Cultural Capital Theory of Pierre Bourdieu, 2024) family cultural capital, as a critical dimension of overall cultural capital, exerts a profound influence on students’ lifestyles, dietary preferences, and bodily awareness by transmitting cognitive frameworks and social dispositions acquired during developmental stages. A study published in 2012 underscored the dual influence of material and cultural capital on dietary patterns, demonstrating that individuals from more affluent families tend to consume greater quantities of healthful foods such as fruits and vegetables, and that cultural capital is significantly correlated with the intake of diverse food categories, including fruits, vegetables, sweets, and sugar-sweetened beverages (Fismen et al., 2012). Furthermore, the cultural milieu fostered by the family predicts the formation of stable eating habits among students, with dynamic elements such as family structure and interaction patterns potentially operating as protective mechanisms that positively guide dietary decision-making (da-Silva-Domingues et al., 2024). A 2020 meta-analysis involving 1,132 college students found that approximately 13.9% experienced varying degrees of disordered eating, suggesting that the family environment may play a pivotal role in shaping and moderating healthy eating behaviors (Chan et al., 2020). According to the Tripartite Influence Model (van den Berg et al., 2002), students’ eating behaviors are significantly influenced by three primary socialization agents—parents, peers, and media—with parental influence exerting particularly enduring effects. Although university life is often regarded as a transitional phase toward autonomy, empirical findings indicate that parental support remains a critical determinant of maladaptive eating patterns, often surpassing the impact of peer influence (Kirsch et al., 2016). This underscores the sustained regulatory role of the family environment in students’ dietary practices. Moreover, familial eating dynamics and financial conditions are closely linked to the implementation of health-oriented dietary strategies, where constructive family discussions about food are associated with reduced intake of high-fat foods and increased consumption of dietary fiber (Schmied et al., 2014). Besides, meta-analysis evidence suggests that this level of correlation is not trivial, but rather quite common in behavioral research (Weinerová et al., 2022; Cohen, 2013). These results indicate that family support is an important (but not the only) contextual resource for the development of healthy eating behaviors among college students, playing a role in conjunction with emotional, cognitive, and situational factors. Collectively, these insights reinforce the conclusion that FS is positively associated with the promotion of healthy eating behaviors among university students, thereby empirically validating hypothesis H1.

The present investigation demonstrates that FS serves as a significant negative predictor of negative emotional states, a finding that echoes prior empirical observations (Lei et al., 2023). Under the moderating influence of this key social support system, university students appear less susceptible to anxiety and depressive symptoms when confronted with daily life stressors. This buffering effect extends beyond the immediate provision of emotional reassurance, likely operating through long-term ecological variables such as familial interaction patterns, access to psychosocial resources, and the educational climate that collectively foster psychological adaptability and promote healthier eating behaviors. According to Bronfenbrenner’s Ecological Systems Theory (Guanghui, 2024), individual development emerges from the dynamic interplay between personal characteristics and the multi-layered environmental contexts. In this framework, students’ emotional states are shaped by proximal influences (e.g., family members, peer relationships) and more distal contextual factors (e.g., socioeconomic status, parental occupation, and cultural heritage). Prior findings have highlighted the critical role of familial communication styles in moderating depressive tendencies among adolescents, with dialogic communication fostering emotional expressiveness and reducing feelings of neglect, thereby mitigating depressive symptoms. In contrast, conformist communication patterns may suppress emotional processing and intensify the accumulation of negative affect (Zhou et al., 2023). The current research further indicates that FS not only influences affective states but also contributes to the formation of attributional styles. As posited by cognitive models, attributional biases play a pivotal role in both the emergence and persistence of anxiety and depression (Wang, 2022). For students exhibiting heightened anxiety, familial care and expectations may paradoxically be perceived as external sources of pressure, amplifying anticipatory concerns and exacerbating anxious responses (Wang et al., 2017). Under conditions of pronounced academic stress, such cognitive interpretations may intensify the perceived impact of FS on anxiety levels. Depressive students, by contrast, often exhibit restricted emotional expressiveness and limited help-seeking behavior, which may hinder their ability to perceive or effectively utilize the emotional resources offered by familial support (Hood et al., 2020). In such cases, deeply entrenched negative cognitive schemas—widely regarded as maintenance mechanisms of depressive symptomatology (Yzer et al., 2022)—may resist alteration, rendering familial support less effective. While the connection between anxiety and disordered eating behaviors has received considerable scholarly attention, the interference mechanisms by which negative emotional states disrupt the formation of healthy dietary patterns remain less explored. Drawing on Affective Regulation Theory (Macht and Simons, 2011), eating behaviors may serve as a compensatory strategy for managing negative emotions, wherein students under heightened psychological strain may gravitate toward high-sugar and high-fat foods to achieve rapid psychological relief. A 2021 cross-sectional study conducted across two Hong Kong universities involving 424 students reported an emotional eating prevalence of 14.8% among female students and 4.5% among males, underscoring the emotional underpinnings of eating behaviors (Sze et al., 2021). Furthermore, nutritional composition can exert bidirectional influences on affective states; deficiencies in key micronutrients such as iron and zinc or in essential vitamins may disrupt neurotransmitter synthesis, thereby precipitating mood dysregulation (Baldwin and Rudge, 1995). In states of emotional distress, students are more likely to opt for calorie-dense, nutritionally sparse food options as a form of short-term psychological compensation, which may ultimately foster maladaptive eating patterns (Eby and Eby, 2006). It is also worth noting that anxiety and depression are associated with distinct neurophysiological pathways and endocrine profiles. FS may alleviate anxiety more directly through physiological mechanisms such as modulation of the hypothalamic–pituitary–adrenal (HPA) axis (Wang et al., 2023). Taken together, these empirical and theoretical insights collectively support hypothesis H2, suggesting that FS ameliorates eating behaviors among university students, in part, by attenuating negative emotional states.

The findings derived from this inquiry indicate that FS exerts a significantly positive predictive effect on university students’ SWB, a conclusion that aligns with existing empirical literature (Zhu et al., 2022). Within the broader framework of emotional regulation and mental health preservation, the emotional bonding and socio-relational resources afforded by familial support function as critical protective factors. According to Personality Trait Theory—Practical Psychology (n.d.), personality characteristics constitute pivotal predictors of SWB; individuals exhibiting high levels of extraversion tend to experience more frequent positive affect and enhanced social functioning, thereby reinforcing their sense of wellbeing, whereas those with elevated neuroticism are more prone to emotional instability, anxiety, and depression, which collectively undermine subjective happiness. Family cultural traits—particularly value orientations emphasizing intellectual and cultural development—have been observed to moderate these relationships. A 2022 study involving 340 Chinese university students reported that familial cultural values, as a complementary dimension of the broader sociocultural system, significantly modulate SWB, and their interaction with personality traits partially accounts for individual variations in wellbeing (Li et al., 2022). These findings underscore the dual role of the familial environment: directly enhancing students’ SWB and indirectly shaping emotional experiences through the formation of personality traits. Perceived FS has been empirically validated as a significant predictor of multiple dimensions of SWB, including life satisfaction, affective stability, and the prevalence of positive emotional states (Brannan et al., 2013). Concurrently, heightened levels of SWB have been closely associated with healthier dietary practices. One investigation involving 73 undergraduate psychology students identified an indirect yet significant link between different components of SWB and dietary quality, whereby individuals with greater wellbeing demonstrated stronger self-regulation and a preference for balanced, nutritious food choices (Jackson and DiPlacido, 2020). Collectively, these empirical and theoretical considerations substantiate Hypothesis H3, which posits that FS facilitates the formation of healthy eating behaviors among university students, in part through the enhancement of SWB.

The results of the present investigation demonstrate a significant inverse relationship between negative affect and SWB among university students, thereby confirming the suppressive influence of negative emotions on the development of wellbeing—an observation consistent with previous empirical findings (Casanova et al., 2023). Drawing upon Cognitive Behavioral Theory (Kalodner, 2011), emotional states are largely shaped by individuals’ cognitive appraisals and attributional styles. When confronted with academic setbacks, students who internalize irrational beliefs—such as “I am incompetent” or “My future is hopeless”—are especially susceptible to anxiety, self-blame, and despondency, all of which substantially undermine their life satisfaction and emotional stability, resulting in diminished SWB. Interventions aimed at correcting cognitive distortions and enhancing cognitive flexibility have been identified as effective mechanisms for disrupting the cyclical relationship between negative affect and lowered wellbeing; structured cognitive training programs designed to help students identify and reframe maladaptive thought patterns have proven successful in mitigating emotional distress and promoting psychological flourishing (Gautam et al., 2020). Moreover, the immune system stress-response hypothesis (Edwards et al., 2007) posits that the consequences of negative affect extend beyond the psychological domain, eliciting physiological stress pathways. During episodes of anxiety or depression, sympathetic nervous system activity is elevated, leading to heightened secretion of stress hormones—such as adrenaline and noradrenaline—from the adrenal medulla. These hormones interact with immune cell receptors, dysregulating immune function and contributing to broader physiological instability. This interplay between emotional dysregulation and physiological stress responses further compromises students’ satisfaction with life and capacity for positive affect, thereby inflicting a dual blow to SWB (D’Acquisto, 2017). High levels of perceived FS serve as a buffering factor within this context, bolstering students’ sense of self-efficacy and enabling greater emotional regulation and psychological resilience in the face of academic and environmental stressors, thereby reducing anxiety levels (Chang et al., 2018). Numerous empirical studies have corroborated the robust association between heightened anxiety and lower levels of SWB across diverse contexts (Liu and Wang, 2024). Taken together, these theoretical and empirical insights lend compelling support to Hypothesis H4, which posits that negative affect functions as a significant negative predictor in the formation of SWB among university students.

This study, based on a university student sample, constructed and empirically validated a chained mediation model exploring the interrelationships among FS, negative emotions, eating behaviors, and SWB, demonstrating that higher levels of perceived FS significantly alleviate negative emotional states while simultaneously fostering healthier dietary habits; moreover, it was found that negative emotions not only directly impair SWB but also indirectly diminish it by exerting adverse effects on eating behaviors, whereas positive dietary patterns serve as a crucial moderating factor in this relationship by buffering the psychological strain induced by emotional fluctuations, thereby contributing to the stabilization of wellbeing; theoretically, these findings extend existing knowledge on the interactive mechanisms between social support and mental health by identifying eating behaviors as a vital regulatory variable, thus enriching the applicability of ecological systems theory and cognitive-behavioral theory within the context of college students’ psychological adaptation, while the combined mediation effect of negative emotion and the buffering role of dietary habits reveal a dynamic linkage between individual behavioral routines and emotional regulation systems, offering a multidimensional framework for understanding the formation of SWB; from a practical standpoint, the results emphasize the foundational role of FS within mental health intervention systems in higher education, suggesting that enhancing students’ wellbeing requires improving the quality of family communication and perceived support while incorporating evidence-based guidance on eating behaviors to achieve dual optimization of emotional and behavioral regulation, and future research should expand the dimensions of social support by investigating the interactive influences of peer relationships and institutional resources, while employing cross-cultural perspectives and longitudinal methodologies to systematically examine the dynamic evolution of SWB across diverse sociocultural contexts, ultimately contributing to the development of universally applicable and operational theoretical foundations and intervention strategies.

## 5 Research limitations and future directions

Focusing on university students, the research offers a systematic examination of the interconnections among FS, negative emotions, eating behaviors, and SWB, presenting notable theoretical depth and practical relevance, with its core strengths embodied in three key aspects: first, by establishing a structural model that simultaneously incorporates emotional and behavioral pathways, it expands existing perspectives on how FS influences SWB and provides robust, systematic evidence for the mediating roles of emotional regulation and health-promoting behaviors; second, clarified the mediating roles of negative emotions and SWB in the relationship between FS and eating behaviors, thereby elucidating the psychological pathways through which mental states influence health-related behaviors. The findings emphasize that improvements in eating behaviors depend not only on effective emotion regulation but can also be achieved through the guidance and cultivation of positive daily behavioral habits; third, the exclusive focus on university students—a population undergoing critical stages of psychological development and behavioral pattern formation—ensures the practical applicability of the findings in the domains of mental health intervention and lifestyle education within higher education contexts; however, certain limitations merit attention. First, data were collected through self-report questionnaires, which may have introduced certain subjective biases, including social desirability bias and recall bias, potentially affecting the objectivity and validity of the data. Furthermore, the cross-sectional design limits the ability to infer causal relationships between variables. It is recommended that future studies use longitudinal tracking to reveal the dynamic interactions between variables over time. Moreover, the association between FS and eating behaviors was relatively weak (*r* = 0.168), indicating that although FS is important, it accounts for only a limited proportion of the variance in students’ eating behaviors. However, the magnitude of this association is consistent with findings from previous research in social psychology and health psychology, which suggest that health-related behaviors are influenced by multiple psychological, social, and environmental factors. Therefore, the relatively small effect size implies that FS should be regarded as an important contextual factor rather than a primary determinant of eating behaviors. Future studies may incorporate a broader range of psychosocial and environmental variables to more comprehensively capture the complexity of eating behaviors among university students. In addition, due to the use of a cluster convenience sampling method, the study sample was primarily drawn from university students in specific regions, which to some extent limited the geographical and cultural heterogeneity of the sample. Accordingly, future research is recommended to replicate and validate the proposed model in cross-cultural samples to examine its applicability across different sociocultural contexts. And the undifferentiated measurement of negative emotions, which overlooks the potentially distinct pathways through which specific emotional components such as anxiety, depression, or anger may operate, suggesting a need for more granular emotional assessment to refine the explanatory power of the model. Notwithstanding these limitations, the findings offer a valuable empirical foundation for mapping the multidimensional pathways through which social support contributes to psychological wellbeing and indicate that integrating eating behaviors into mental health interventions may represent a promising and underexplored avenue for promoting holistic wellbeing among university populations.

## 6 Conclusion

The results indicate that FS significantly contributes to improved eating behaviors among university students, with negative emotions and SWB jointly constituting a chained mediation pathway. Specifically, FS facilitates the establishment of positive dietary habits by reducing negative emotions, with the mediating effect of negative emotions proving to be considerably stronger than that of SWB, highlighting the critical importance of emotional regulation in this process. The research further suggests that positive family interactions enhance emotional stability, indirectly guiding students toward adopting healthier eating behaviors. This conclusion not only deepens the understanding of the mechanisms linking social support to behavioral habits but also offers practical implications for psychological interventions, underscoring the vital role of familial emotional resources in alleviating negative emotions and optimizing behavioral choices.

## Data Availability

The raw data supporting the conclusions of this article will be made available by the authors without undue reservation.
